# Case Report: Ovarian dysgerminoma mimicking a sex cord-stromal tumor with hyperandrogenism: diagnostic challenges and molecular insights

**DOI:** 10.3389/fonc.2026.1802097

**Published:** 2026-05-15

**Authors:** Yuan Wang, Xue Hao, Jiajun Li, Yifan Gao, Juan Wang, Wenxin Wu, Jinfeng Cui

**Affiliations:** 1 Department of Pathology, The Second Hospital of Hebei Medical University, Shijiazhuang, China; 2 School of Pharmacy, Hebei Medical University, Shijiazhuang, China; 3 School of Basic Medical Sciences, Hebei Medical University, Shijiazhuang, China

**Keywords:** diagnostic challenge, functioning stroma, hyperandrogenism, KRAS and CDK4 amplifications, mimicking a sex cord-stromal tumor, ovarian dysgerminoma

## Abstract

Ovarian dysgerminoma, a malignant germ cell tumor, rarely presents with endocrine manifestations. This report describes a rare case of an 8-year-old girl with virilization symptoms and markedly elevated serum testosterone, initially suggestive of a steroid-producing sex cord-stromal tumor. Histopathological examination revealed a dysgerminoma with highly unusual architectural patterns, including pseudoglandular, follicular, and tubular structures, along with prominent luteinized stromal cells. Immunohistochemistry was diagnostic, showing germ cell markers (SALL4+, OCT3/4+, CD117+) and β-HCG-positive syncytiotrophoblasts associated with inhibin-α-positive stroma, thus explaining the androgen excess. Molecular profiling revealed concurrent *KRAS* and *CDK4* amplifications, providing a plausible mechanistic basis for the tumor’s high proliferative index and aggressive behavior, and underscoring the biological heterogeneity underlying this rare presentation. The patient achieved sustained remission after surgery and chemotherapy during a follow-up period of five years and six months. This case underscores the significant diagnostic challenge posed by dysgerminomas mimicking sex cord-stromal tumors and highlights the critical role of an integrated diagnostic approach combining morphology, immunohistochemistry, and molecular profiling to avoid misdiagnosis and guide management.

## Introduction

The diagnosis of ovarian neoplasms in children and adolescents can be particularly challenging when clinical and pathological features point in contradictory directions. Ovarian dysgerminoma is a malignant ovarian germ cell tumor, accounting for 1% to 2% of all malignant ovarian neoplasms. It predominantly affects adolescents and young adults, with cases in prepubertal children being particularly rare (1, 2). Classically, ovarian dysgerminoma tumor cells are arranged in nests or sheets, separated by fibrous stroma that often contains scattered lymphocytes (3). In addition, endocrine manifestations are highly atypical for dysgerminoma, as they are not derived from steroidogenic cells (4). However, we present a case that defies this typical presentation, creating a significant diagnostic dilemma. An 8-year-old girl presented not only with a large ovarian mass but also with clinical and biochemical evidence of profound hyperandrogenism, a finding exceptionally uncommon in dysgerminoma. Compounding the diagnostic difficulty, the tumor exhibited unusual morphological patterns, including pseudoglandular, follicular, and tubular architectures, which are classic mimics of sex cord-stromal tumors. This convergence of rare endocrine activity with deceptive morphology underscores a major diagnostic pitfall. While dysgerminomas are generally genomically stable, emerging evidence of molecular heterogeneity, including MAPK pathway alterations, may offer new insights into the pathogenesis of such rare phenotypic variants (5). This case highlights the critical importance of integrating clinical context, sophisticated histopathological evaluation, and molecular profiling to accurately diagnose complex pediatric ovarian tumors.

## Case presentation

An 8-year-old girl presented with a one-week history of abdominal distension, abdominal pain, occasional nausea, vomiting, and vaginal bleeding. She was initially diagnosed with intestinal obstruction at a local hospital, but treatment for this condition was ineffective. The patient had experienced menarche two months earlier, with menstrual periods lasting 3–4 days, suggesting possible precocious puberty. Further medical history review revealed symptoms suggestive of hyperandrogenism, including voice deepening and significant weight gain over the previous two months. Her past medical history and family history were unremarkable.

**Figure 1:**
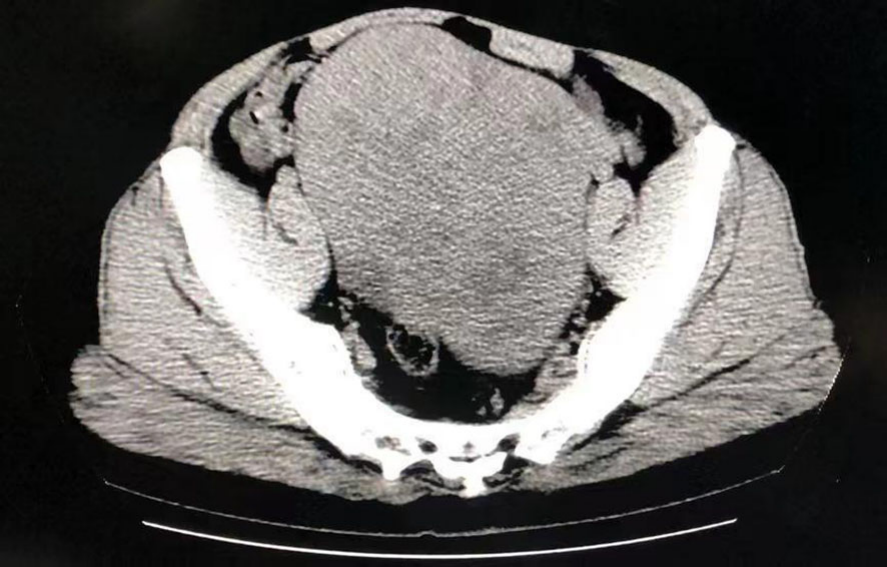
Computed tomography obtained showing space-occupying lesions in the lower abdomen.

Physical examination revealed a distended, firm, and tender abdomen, with positive shifting dullness on percussion. Gynecological examination showed a moderately developed vulva with clitoral hypertrophy and a small amount of pubic hair. Abdominal CT (**Figure 1**) revealed a large, solid-cystic mass measuring approximately 12.5 × 10.8 cm occupying the lower abdomen and pelvic cavity, originating from the right adnexal region. The mass displaced the bowel loops superiorly, with no clear evidence of direct invasion into the bladder or rectum. Massive abdominal and pelvic ascites was also present. Given the patient’s advanced pubertal development, including menarche and clitoral hypertrophy, transabdominal ultrasound was initially performed, followed by transvaginal ultrasonography (TVS) to obtain higher-resolution imaging for preoperative evaluation. Transvaginal ultrasonography (TVS) identified pelvic and abdominal masses consistent with ovarian lesions, also confirming the presence of ascites. Uterine size was discrepant for the patient’s age. Sex hormone analysis showed a significantly elevated testosterone level (>16.00 ng/ml), exceeding the normal reference range (0.00–0.75 ng/ml). Other hormones, including follicle-stimulating hormone (FSH), luteinizing hormone (LH), estradiol, progesterone, and serum prolactin, were within normal limits. Tumor marker analysis revealed a markedly elevated serum CA-125 level of 248 U/mL (normal <35 U/mL), while AFP, CEA, CA19-9, and CA15–3 were within normal range.

The patient was evaluated by a pediatric endocrinologist and a geneticist. Genetic counseling was offered; however, in the absence of clinical features or family history indicative of a disorder of sex development or a hereditary tumor syndrome, germline genetic testing was not pursued.

The patient underwent exploratory laparotomy with right adnexectomy, left ovarian biopsy, and multipoint biopsies of the peritoneum and greater omentum under general anesthesia. Approximately 4,200 ml of ascitic fluid was aspirated during the procedure.

**Figure 2:**
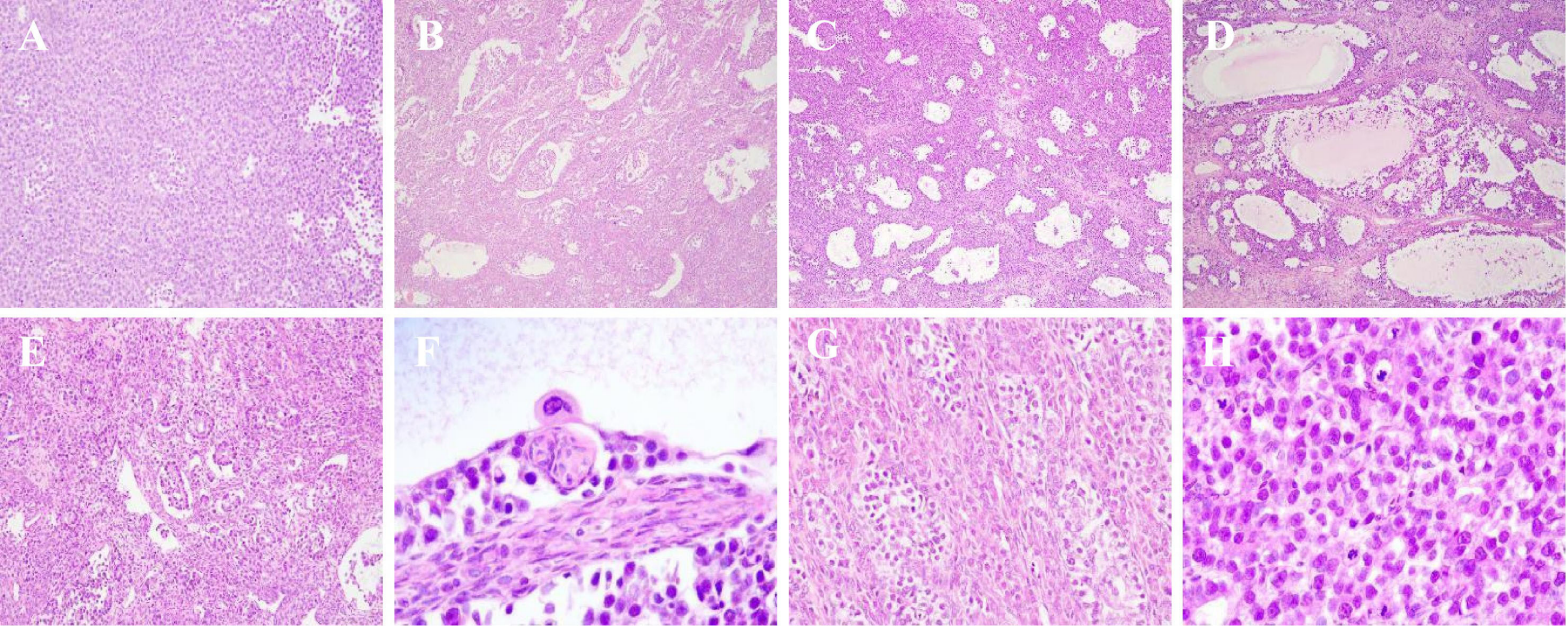
Histopathological examination showed sheet **(A)** and nests **(B)** structure, pseudoglandular tubular structure **(C)**, follicular structures **(D)**, and some tubular structures which are similar to sertoli cell tumors **(E)** (×100). Besides, syncytiotrophoblasts **(F)** can be observed and the presence of mitosis in tumor cells is more frequent under high magnification **(G)** (×400). The luteinized cells cytoplasm exhibits a high degree of eosinophilia **(H)** (×200) in tumor stroma.

Gross examination of the right ovary revealed a 12 × 10 × 7 cm mass with a solid, grayish-yellow appearance and soft texture. Microscopically, the tumor tissue grew in sheets and nests (**Figures 2A, B**). Unusual architectural patterns were also observed, including pseudoglandular tubular and follicular structures (**Figures 2C, D**), as well as tubular structures resembling those seen in sex cord-stromal tumors (**Figure 2E**). In some areas, the cytoplasm of the tumor interstitial cells appeared reddish. High-power examination showed that the tumor cells were uniform in morphology, polygonal in shape, with abundant, slightly eosinophilic cytoplasm, vacuolated nuclei, and prominent nucleoli. Scattered syncytiotrophoblasts were also observed within the tumor on paraffin sections (**Figure 2F**). Mitotic figures were frequently identified (**Figure 2G**). The tumor stroma contained cells with highly eosinophilic cytoplasm, resembling Leydig cells, identified as luteinized cells (**Figure 2H**). These luteinized cells were present within the stroma alongside occasional lymphocytes.

**Figure 3:**
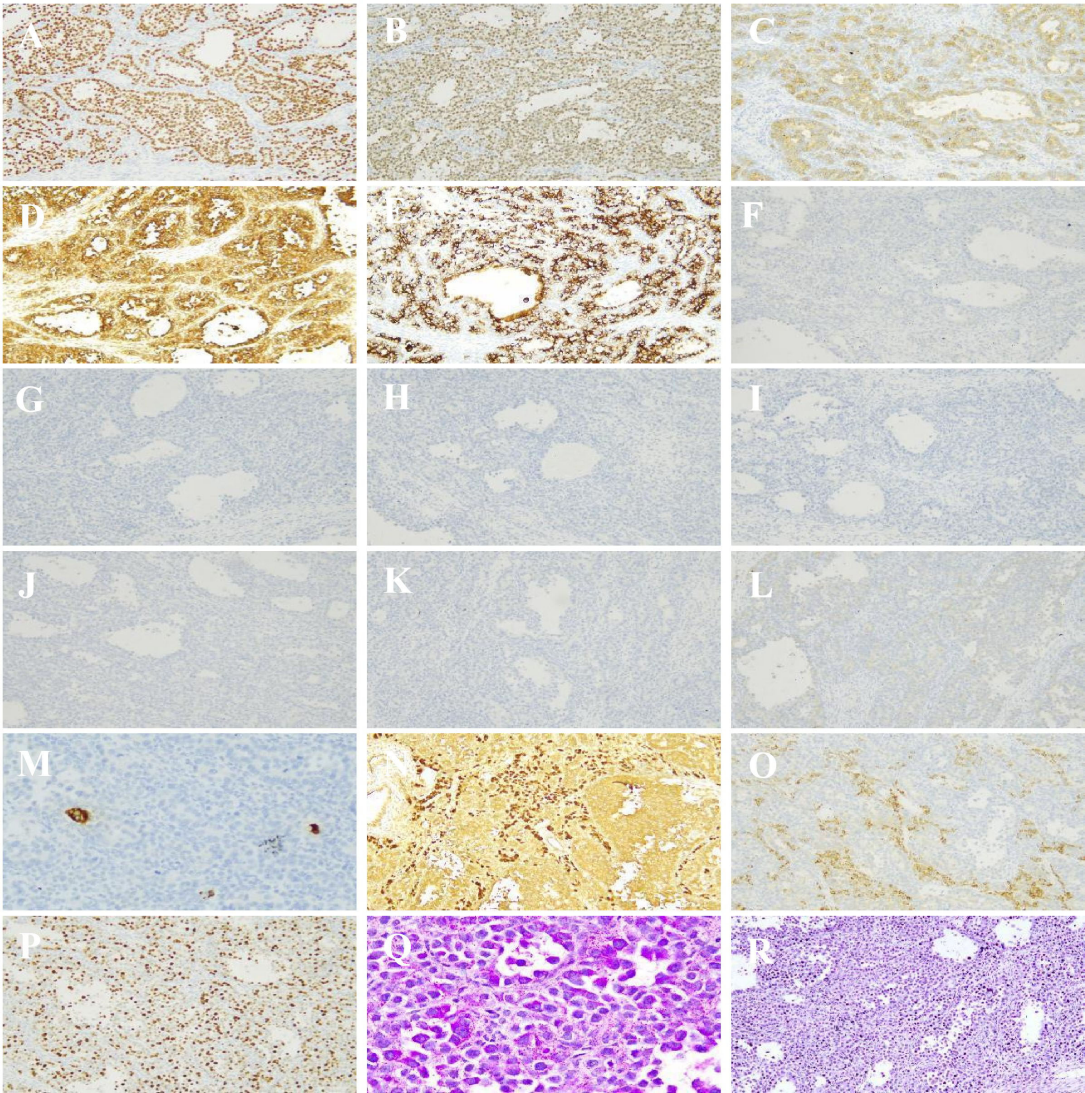
Immunohistochemical staining revealed positive expression of SALL4 **(A)**, OCT3/4 **(B)**, CD117 **(C)**, D2-40 **(D)** and AE1/AE3 **(E)**, but negative for Glypican-3 **(F)**, CD30 **(G)**, CD56 **(H)**, PLAP **(I)**, WT-1 **(J)**, AFP **(K)** and CK7 **(L)**. β-HCG exhibited cytoplasm immunoreactivity for the scattered syncytiotrophoblast cells **(M)**. In addition, the interstitial cells partially expressed Calretinnin **(N)** and Inhibin-α **(O)**. The Ki-67 positive index reached 60% in the area of the greatest concentration **(P)**. PAS staining showed positive red grain in the cytoplasm of tumor cells **(Q)**, and digestive PAS was negative **(R)** (×100).

Immunohistochemistry (IHC) staining showed that the tumor cells were strongly positive for SALL4 (**Figure 3A**), OCT3/4 (**Figure 3B**), CD117 (**Figure 3C**), D2-40 (**Figure 3D**), and AE1/AE3 (**Figure 3E**), but negative for Glypican-3 (**Figure 3F**), CD30 (**Figure 3G**), CD56 (**Figure 3H**), PLAP (**Figure 3I**), WT-1 (**Figure 3J**), alpha-fetoprotein (AFP) (**Figure 3K**), and CK7 (**Figure 3L**). β-HCG showed cytoplasmic immunoreactivity in the scattered syncytiotrophoblast cells (**Figure 3M**). The interstitial cells were partially positive for Calretinin (**Figure 3N**) and Inhibin-α (**Figure 3O**). The Ki-67 proliferation index reached 60% in the most concentrated areas (**Figure 3P**). PAS staining revealed positive red cytoplasmic granules in the tumor cells (**Figure 3Q**), which were digested by diastase (**Figure 3R**), confirming the presence of glycogen.

**Figure 4:**
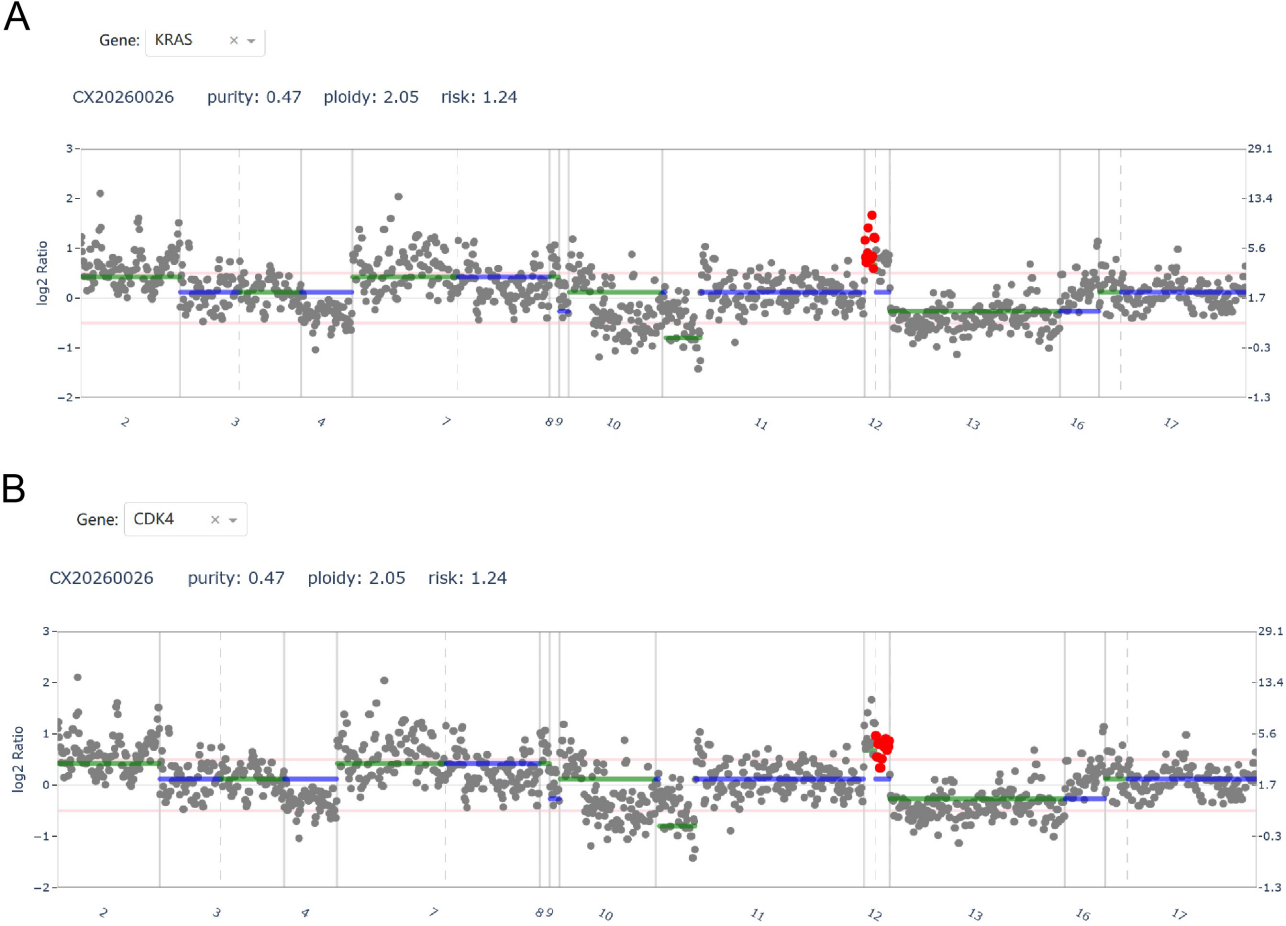
Schematic representation of genomic alterations detected by high-throughput next-generation sequencing. The tumor harbored amplifications in *KRAS* **(A)** and *CDK4* **(B)**.

To explore the potential molecular mechanisms underlying this rare case, high-throughput next-generation sequencing was performed on the tumor tissue. The microsatellite instability (MSI) status was determined to be stable (MSS). Genomic variant analysis revealed two somatic amplifications: *KRAS* with a copy number (CN) of 4.2 (**Table 1**; **Figure 4A**) and *CDK4* with a CN of 3.4 (**Table 1**; **Figure 4B**). No other pathogenic mutations or fusions were detected within the tested gene panel.

**Table 1:** Summary of key molecular pathological findings in the ovarian dysgerminoma.

Gene	Result/transcript	Functional region	Variant type	Mutant abundance
*KRAS*	NM_033360.2	all exon	Amplification	CN:4.2
*CDK4*	NM_000075.3	all exon	Amplification	CN:3.4

## Diagnosis and clinical course

In summary, based on serum hormone levels, histological morphology, immunophenotype, PAS staining, and molecular genetic results, the final diagnosis was “dysgerminoma with androgen-producing functioning stroma (right ovary).

Postoperatively, the patient’s testosterone and CA-125 levels returned to normal, and the ascites resolved. She received four cycles of adjuvant chemotherapy with paclitaxel and carboplatin. Postoperative follow-up consisted of clinical examination, serum tumor markers (AFP, β-hCG, CA-125, and testosterone), and abdominopelvic ultrasound every 3 months for the first 2 years, then every 6 months up to 5.5 years. All results remained within normal limits, and there was no evidence of recurrence during this period.

## Discussion

Dysgerminoma is a malignant tumor derived from ovarian primordial germ cells and is most common in young women. Early-stage dysgerminomas often lack specific symptoms and are frequently detected only when the tumor becomes large enough to cause compression of surrounding tissues, leading to abdominal pain, bloating, or ascites. Imaging studies are useful for auxiliary diagnosis but often lack specificity, making histopathological examination essential for definitive diagnosis. The histopathological diagnosis of dysgerminoma, like its testicular counterpart seminoma, is usually straightforward due to its classic appearance: tumor cells with pale to clear cytoplasm arranged in nests separated by delicate fibrous septa infiltrated by lymphocytes (3). While the vast majority of dysgerminomas are “non-functioning,” significant diagnostic challenges arise when they exhibit rare histological variants or functioning stroma, leading to aberrant endocrine manifestations (6, 7). This report describes an exceptionally rare case of ovarian dysgerminoma in an 8-year-old girl presenting with hyperandrogenism (virilization) and precocious puberty. The tumor mimicked a sex cord-stromal tumor morphologically and harbored atypical co-amplifications of *KRAS* and *CDK4*. This case underscores the importance of an integrated diagnostic approach and provides novel insights into the clinicopathological and molecular heterogeneity of this tumor.

## Histopathological and clinical characteristics

The classic morphology of ovarian dysgerminoma is well-defined (3). However, a broad morphological spectrum has been well-documented in the literature, including corded, trabecular, microcystic, and even pseudoglandular or tubular patterns. These atypical features can closely mimic sex cord-stromal tumors such as Sertoli cell tumor, posing a diagnostic pitfall (6, 8). More rarely, approximately 5% of cases contain syncytiotrophoblastic giant cells (STGCs) that produce human chorionic gonadotropin (hCG). The secreted hCG may act in a paracrine fashion to stimulate luteinization of the tumor stromal cells, forming so-called “functioning stroma,” which can subsequently lead to abnormal secretion of steroid hormones like estrogen or androgen (7, 9).

The present case exemplifies these rare features. Histologically, in addition to the typical solid and nested growth pattern, the tumor exhibited prominent pseudoglandular and tubular structures, closely simulating a sex cord-stromal tumor. The crucial finding was the presence of numerous luteinized cells within the tumor stroma. This morphology directly correlated with the striking clinical presentation: an 8-year-old girl with virilization signs (voice deepening, significant weight gain) and markedly elevated serum testosterone levels.

A review of the literature confirms that dysgerminomas with endocrine manifestations are exceedingly rare (**Table 2**). The clinical spectrum includes estrogenic precocious puberty (4, 10) and androgenic virilization (11). The pathogenesis can be mediated by hCG from STGCs (10) or, in the absence of hCG, by aberrant expression of steroidogenic enzymes (e.g., P450 aromatase) within the stromal cells themselves (4). As illustrated in **Table 2**, the co-occurrence of virilization and precocious puberty in our patient represents a particularly unique presentation among reported cases (4, 10–12).

**Table 2:** Representative Reported Cases of Ovarian Dysgerminoma with Functioning Stroma and Associated Endocrine Manifestations.

Reference	Age	Key clinical presentation	Hormonal change	Key pathological clues	Follow-up
Ueda et al. (1972) (10)	22 years old	Amenorrhea, Hirsutism	↑ Urinary 17-KS	Contains STGCs (hCG+); Leydig-like cells in stroma	N/A
Gücer et al. (2005) (11)	25 years old	Pseudo-Meigs Syndrome, Ascites	↑ Free Testosterone, Androstenedione	Stromal luteinization; Tumor cells PLAP+; No STGCs identified	NED, 12 months
Song et al. (2007) (12)	8 years old	Precocious Puberty	↑ Estradiol	Contains STGCs (hCG+); Stroma: ER/PR+	NED, 24 months
Nagase et al. (2021) (4)	7 years old	Precocious Puberty	↑ Estradiol	Stroma expresses SF-1, P450arom, etc. (No STGCs)	NED, >24 months
The present study	8 years old	Virilization, Precocious Puberty	↑ Testosterone	Contains STGCs (β-hCG+); Stroma: Inhibin-α+/Calretinin+; KRAS/CDK4 amplification	NED, 66 months

NED, No Evidence of Disease; STGCs, Syncytiotrophoblastic Giant Cells.

## Diagnostic challenge and integrated diagnostic approach

The principal diagnostic dilemma in this case was distinguishing an ovarian dysgerminoma with morphology mimicking a sex cord-stromal tumor from a true hormone-producing sex cord-stromal tumor (e.g., Sertoli-Leydig cell tumor). This diagnostic difficulty is well-recognized in pathological practice when confronting atypical morphological patterns (6, 8).

In this context, immunohistochemistry (IHC) serves as an indispensable diagnostic “gold standard.” In our case, the tumor cells showed diffuse and strong positivity for germ cell-specific markers SALL4, OCT3/4, and CD117. In contrast, sex cord-stromal markers (Inhibin-α, Calretinin) were expressed only in the reactive luteinized stromal cells, with the tumor cells themselves being negative. This distinct immunophenotype unequivocally confirmed the germ cell origin.

The pathological findings in this case also support the classic pathogenetic hypothesis of functioning stroma: the scattered β-hCG-positive STGCs within the tumor stimulated the surrounding ovarian-type stroma to luteinize and express Inhibin-α via a paracrine mechanism, leading to the overproduction of androgen (testosterone) (7, 10). This mechanism effectively links the morphology, immunophenotype, and clinical presentation.

In addition to dysgerminoma with atypical morphology, other ovarian and extra-ovarian neoplasms may also histologically mimic sex cord-stromal tumors. These include juvenile granulosa cell tumors, Sertoli-Leydig cell tumors, and certain yolk sac tumors with prominent stromal components. Moreover, in pediatric and adolescent populations, the differential diagnosis of pelvic masses should also consider benign entities such as mature teratomas and functional ovarian cysts. A multidisciplinary approach integrating imaging, serum tumor markers, and hormone levels is essential for guiding surgical strategy and avoiding unnecessary oophorectomy. Recent studies emphasize the importance of risk-stratified management to preserve fertility while ensuring oncologic safety. Furthermore, the classic triad of pelvic mass, ascites, and pleural effusion with elevated CA-125 levels—as seen in Meigs syndrome—can mimic malignancy and should be considered in the differential diagnosis of ovarian tumors presenting with ascites. Furthermore, the classic triad of pelvic mass, ascites, and pleural effusion with elevated CA-125 levels—as seen in Meigs syndrome—can mimic malignancy and should be considered in the differential diagnosis of ovarian tumors presenting with ascites (13). In the present case, the markedly elevated serum CA-125 (248 U/mL) was attributed to peritoneal irritation from the massive ascites and large pelvic mass, akin to mechanisms observed in Meigs’ syndrome; the level normalized rapidly after tumor resection, further supporting a reactive rather than neoplastic etiology of this marker elevation.

## Molecular pathogenesis, therapeutic implications, and prognostic context

On a molecular level, ovarian dysgerminomas are generally genomically stable. Characteristic alterations include frequent KIT gene mutations (found in approximately 25-33% of cases) and sustained high expression of pluripotency markers like OCT3/4 (14). These features constitute the classic molecular profile.

In this case, high-throughput sequencing revealed co-amplifications of *KRAS* (copy number 4.2) and *CDK4* (copy number 3.4). This molecular event is not typical of classic dysgerminoma. *KRAS* is a key driver of the MAPK signaling pathway, and its amplification may lead to constitutive pathway activation, promoting cell proliferation. *CDK4* is a crucial regulator of the cell cycle, and its amplification can drive cell cycle progression. The synergistic effect of these two amplifications may partly explain the high proliferative index (Ki-67 60%) observed in this tumor. Although atypical, this finding suggests the possible existence of unrecognized molecular subtypes of dysgerminoma, where distinct genetic alterations might be associated with special clinicopathological phenotypes, such as prominent endocrine activity—a hypothesis that warrants further investigation.

Regarding treatment and prognosis, ovarian dysgerminomas respond excellently to standard chemotherapy regimens. The overall survival rate exceeds 90% for early-stage patients treated with surgery and platinum-based adjuvant chemotherapy (3). Our patient underwent unilateral adnexectomy followed by adjuvant paclitaxel/carboplatin chemotherapy and achieved long-term disease-free survival (>66 months). This outcome aligns with the generally favorable prognosis of this disease, indicating that even with a dramatic clinical presentation and atypical molecular findings, sensitivity to standard treatment and a good final outcome are maintained.

It is important to note that this study did not include germline genetic testing for conditions predisposing to germ cell tumors, such as Turner syndrome, Swyer syndrome, or other disorders of sex development. While the patient had no clinical features suggestive of an underlying syndrome, the possibility of a genetic predisposition cannot be entirely excluded. Future studies incorporating germline analysis may provide further insight into the pathogenesis of such rare cases.

## Conclusion and future perspectives

This case significantly expands the recognized clinicopathological and molecular spectrum of ovarian dysgerminoma. It reinforces several critical points: First, dysgerminoma must remain a key consideration in the differential diagnosis of hormonally active pelvic masses in young patients, even when morphology strongly suggests a sex cord-stromal tumor. Second, a systematic, integrated diagnostic workflow progressing from histomorphology to targeted IHC (germ cell vs. sex cord-stromal markers) is essential. Third, the discovery of *KRAS*/*CDK4* co-amplification, though non-classical, highlights the underlying molecular heterogeneity of this tumor and suggests that specific clinical manifestations may be linked to particular genomic events.

Future research on such rare variants should focus on: 1) Establishing multicenter collaborative networks to systematically collect clinical, pathological, and molecular data; 2) Conducting more comprehensive genomic studies to delineate the complete molecular landscape and explore genotype-phenotype correlations; 3) Considering the incorporation of molecular testing into the diagnostic workflow for atypical cases, which may provide clues for potential personalized therapeutic strategies in the future.

## Data Availability

The original contributions presented in the study are included in the article/supplementary material. Further inquiries can be directed to the corresponding author.
